# First principles study of electronic and optical properties and photocatalytic performance of GaN–SiS van der Waals heterostructure

**DOI:** 10.1039/d1ra06011b

**Published:** 2021-10-07

**Authors:** S. S. Ullah, M. Farooq, H. U. Din, Q. Alam, M. Idrees, M. Bilal, B. Amin

**Affiliations:** Department of Physics, Hazara University Mansehra Pakistan; Department of Physics, Abbottabad University of Science and Technology Abbottabad 22010 Pakistan haleem.uddin@yahoo.com; Department of Physics, Bacha Khan University Charsadda Pakistan

## Abstract

The vertical stacking of two-dimensional materials *via* van der Waals (vdW) interaction is a promising technique for tailoring the physical properties and fabricating potential devices to be applied in the emerging fields of materials science and nanotechnology. The structural, electronic and optical properties and photocatalytic performance of a GaN–SiS vdW heterostructure were explored using first principles calculations. The most stable stacking configuration found energetically stable, possesses a direct staggered band gap, which is crucial for separating photogenerated charged carriers in different constituents and is efficacious for solar cells. Further, the charge transfer occurred from the SiS to GaN layer, indicating that SiS exhibits p-type doping in the GaN–SiS heterobilayer. Interestingly, a systematic red-shift was observed in the optical absorption spectra of the understudy heterobilayer system. Moreover, the conduction band edge and valence band edge of the monolayers and corresponding heterostructure were located above and below the standard redox potentials for photocatalytic water splitting, making these systems promising for water dissociation for hydrogen fuel production. The results provide a route to design the GaN–SiS vdW heterostructure for the practical realization of next-generation light detection and energy harvesting devices.

## Abstract 2

The two dimensional GaN–SiS van der Waals heterostructure is a promising candidate for optoelectronic and photocatalytic water splitting.

## Graphical abstract

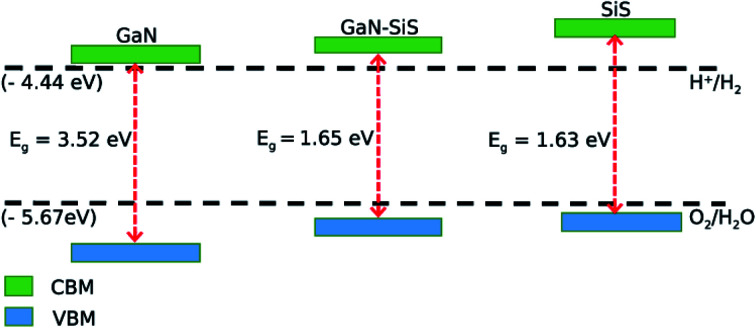

## 1. Introduction

Hydrogen with one electron and one proton is found in the earth's crust.^1^ It can be produced from other key energy sources through an energy conversion process.^2^ It is a secondary source of renewable energies and plays a critical role in developing environmentally friendly, low-emission, renewable and long-term energy systems.^3^ Hydrogen and fuel cells have a wide variety of possible applications in transportation, commerce, business, residential and portable applications. The technology of hydrogen fuel cells is currently being explored. This type of technology allows for the development of electrical energy from hydrogen, which is a pollution-free energy source that can be used to power automobiles. When hydrogen is burned, it creates water, turning it into a pollution-free fuel. However, the process of manufacturing hydrogen fuel is not particularly safe or pollution-free. Currently, natural gas is used to manufacture the majority of hydrogen, which contains carbon dioxide.^4^

Since the earth's surface contains 71% water, hydrogen can be produced through the photocatalytic decomposition of water. Extracting hydrogen from this process is an alternative approach and highly desirable for a clean environment.^5^ It is a promising technology for generating renewable and clean energy, which is pollution-free with no emissions of toxic gases.^6–8^ Also, it has been the subject of research due to its importance in resolving energy crises and environmental issues.^9^ Since semiconductors have chemical and photochemical stability as well as a high absorption capacity of visible light, they can be used for water splitting. Proper valence and conduction band edge orientation make semiconductors suitable to be used for water decomposition.^10^

It is more difficult to choose the best photocatalyst. In general, there are two types of photocatalytic reactions: first, the uphill reaction, in which energy of photons is absorbed by the photocatalyst and transformed into chemical energy, and second, the downhill reaction, in which the photocatalyst absorbs the energy of a photon and uses it to initiate a thermodynamic reaction.^11^ Water splitting produces hydrogen and oxygen, which is an “uphill” reaction. If the energy of the incident light is greater than the band gap, then electrons are created in the conduction band, while holes are created in the valence band of semiconductors. Complete water decomposition occurs because of the reduction and oxidation of water molecules by electrons and holes, respectively.^12^ H^+^/H_2_ redox potential (0 eV) should be smaller than the conduction band minima, while the redox potential of O_2_/H_2_O (1.23 eV) must not be less than the conduction band maxima.^13^ In short, we can say that the band gap of the semiconductor must be greater than 1.23 eV.

Due to their novel properties^14–20^ and wide variety of applications in fields, such as catalysis,^21^ electrochemical energy storage,^22^ electronics,^23^ spintronic^24,25^ and photonic nanodevices,^26^ two-dimensional layered materials are significantly the focus of current research. The migration enhanced encapsulated growth (MEEG) method has been used to fabricate graphene-like gallium nitride (g-GaN).^27^ The monolayer g-GaN semiconductor has a large band gap of about 4 eV.^28^ Strain engineering and stacking heterostructures can enhance the properties of two-dimensional single-layer g-GaN.^29–31^ Furthermore, the photocatalytic application of the heterostructure of g-GaN and BlueP is possible due to the type-II band alignment.^28,32,33^ Also, Ren *et al.*^32^ confirmed that the g-GaN/BSe heterostructure has a type-II band alignment, which can continuously promote the separation of photogenerated charge carriers.

Two-dimensional SiS is ideal for photovoltaic applications because it has tunable electronic properties, indirect band gap, high carrier mobility, mechanically and chemically stability, and versatile anisotropic optical behavior.^34^ Studies showed that materials with an indirect bandgap are better for photocatalytic activity.^35^ SiS has a suitable bandgap value (*E*_g_ > 1.23 eV), ideal positions of the band edges and considerable optical absorption. These properties make it promising to be used in Li-ion batteries as an anode material.^36^ Two-dimensional materials are vertically stacked through van der Waals (vdW) interactions,^37–45^ which lead to the exploration of new phenomena and designing feasible optoelectronic devices such as flexible optoelectronic equipment,^46^ tunnel transistors^47^ and constructive tools.^48^

Three types of band alignments are type-I, type-II, or type-III in the vdW heterostructure, each having its unique application, allowing for the realization of various instruments.^49^ Staggered or type-II band alignment with valence band maximum (VBM) and conduction band minimum (CBM) contributed by two separate constituents will trigger optical excitation between the layers and regulates transition energy between layers. As a result, these materials are promising for the applications of solar cells, and the generation of photovoltaic power because of charge carrier separation is dominated here. Thus, in the development of advanced optoelectronic devices^50^ and photocatalytic applications,^51^ heterostructures with the staggered band alignment have drawn considerable interest. Theoretically and experimentally, lateral heterostructures have been built by in-plane stitching.^52^ However, producing lateral heterostructures having several junctions with high quality is still a challenge.^53^ In the vdW heterostructure, there is no direct chemical bond between atoms. While retaining the interlayer separation required for the vdW interaction, the lattice mismatch conditions can be relaxed to allow different layers with different lattice constants to be stacked.

In this study, we used density functional theory to look at the electronic properties of GaN–SiS vdW heterostructures in various stacking patterns. The lowest binding energy confirms the energetically stable structure among all possible configurations of the GaN–SiS heterobilayer. A direct type-II band alignment is found in the GaN–SiS vdW heterostructures. GaN–SiS vdW heterostructures also have great capability for excellent visible light absorption, which leads to applications in solar cells and optoelectronics. In addition, the understudy monolayers and heterobilayer are capable of photocatalytic water splitting, indicating their good performance of photocatalytic hydrogen fuel production.

## 2. Computational details

The quantum espresso^54,55^ is used to perform first-principles calculations based on density functional theory (DFT).^56^ Electronic exchange and correlation energies are determined using a type of the generalized gradient approximation (GGA)^57^ known as the Perdew–Burke–Ernzerhof (PBE) level.^58^ The Grimme's dispersion correction (DFT-D2)^59^ method was employed for the vdW correction. A vacuum layer of 25 Å was set in the *z*-direction for preventing interactions between adjacent layers. The plane wave kinetic energy cutoff was set at 500 eV. For optimization, a structure having a precision high grid of *k*-points was sampled *via* a *k*-grid integrated into a Brillouin zone centered at 12 × 12 × 1 and geometrically relaxed at 6 × 6 × 1. The geometric relaxation and electronic properties were carried out until the energy and forces converged to value of 10^−5^ eV and 0.001 eV Å^−1^, respectively. Also, HSE06 functional was adopted for a better understanding of the electronic band structure. The GW_o_ method was used to measure the optical absorption spectra of the vdW heterostructure and constituent monolayers. The GW_o_ approach was adapted, where the HSE06 single-particle energies and wave functions were used to calculate the Quasiparticle energies and solve the Bethe–Salpeter equation (TammDancoff approximation). We took into account the 10 highest valence and 10 lowest conduction bands to calculate the excitonic eigenstates.

## 3. Results and discussion

**Fig. 1:**
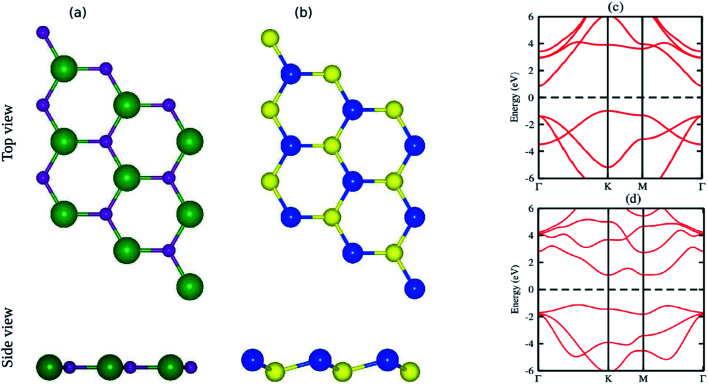
Top and side view of (a) GaN and (b) SiS monolayers, respectively. Light green, light blue, blue and yellow balls represent the Ga, N, Si and S atoms, respectively. Band structures of (c) GaN and (d) SiS monolayers.

The pristine GaN and SiS monolayers have planar and buckled graphene-like honeycomb structural geometries, as displayed in Fig. 1(a and b). The optimized lattice constants of single layer GaN and SiS are 3.25 Å and 3.29 Å, respectively. Using PBE functional, an indirect semiconducting band gap with values of 1.90 eV for GaN and 2.18 eV for SiS was obtained, as shown in Fig. 1(c and d). These results are in good agreement with the available literature,^60–63^ indicating the reliability of the present study (Table 1).

**Table 1:** Lattice constant (*a*), bond length (Ga–N, Si–S), binding energy (*E*_b_), interlayer distance (*d*), band gap (*E*_g_), conduction and valence band edge potentials (*E*_CB_, *E*_VB_) of the GaN, SiS monolayers and the GaN–SiS heterostructure

Parameters	GaN	SiS	GaN–SiS
*a* (Å)	3.25	3.29	3.27
Ga–N (Å)	—	—	1.86
Si–S (Å)	—	—	1.33
Stacking-a, *E*_b_/*d* (eV Å^−1^)	—	—	−0.115/3.542
Stacking-b, *E*_b_/*d* (eV Å^−1^)	—	—	−0.160/3.275
Stacking-c, *E*_b_/*d* (eV Å^−1^)	—	—	−0.163/3.178
Stacking-d, *E*_b_/*d* (eV Å^−1^)	—	—	−0.165/3.106
Stacking-e, *E*_b_/*d* (eV Å^−1^)	—	—	−0.152/3.301
Stacking-f, *E*_b_/*d* (eV Å^−1^)	—	—	−0.110/3.566
*E*_g_ (PBE) (eV)	1.90	2.18	1.48
*E*_CB_ (eV)	−1.5	−0.14	−0.62
*E*_VB_ (eV)	0.34	2.04	0.86

**Fig. 2:**
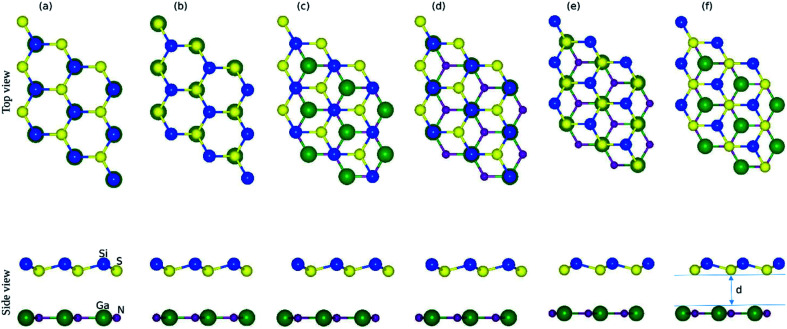
Top view and side view of six possible stacking configurations (a)–(f) of the GaN–SiS vdW heterostructure and “*d*” is the interlayer distance. The details of different geometrical stacking are given in the text.

The small lattice mismatch allowed the fabrication of the vdW heterostructure of GaN and SiS monolayers. As the layer stacking was sensitive to the orientation of individual constituents, six possible stacking configurations of the GaN–SiS vdW heterostructure are presented in Fig. 2. In stacking a(b), the Si atom is placed on top of the Ga(N)-atom and the S atom is fixed above the N(Ga)-atom. For the stacking c(d), Si-atom was positioned above the N(Ga)-atom, while S was located at the hollow site. The S-atom was placed on top of the N(Ga)-atom, while the Si-atom was fixed at the center of the hexagonal.

The difference in total energy between the heterostructure and its parent monolayers is called binding energy (*E*_b_):*E*_b_ = *E*_hetero_ − *E*_i-monolayer_ − *E*_ii-monolayer_

The calculated binding energy (interlayer distance) of GaN–SiS heterostructures for (a), (b), (c), (d), (e), and (f) patterns are −0.115 eV (3.54 Å), −0.160 eV (3.27 Å), −0.163 eV (3.17 Å), −0.165 eV (3.10 Å), −0.152 eV (3.30 Å), and −0.110 eV (3.56 Å), respectively. The stacking (d) possessed the lowest binding energy and a smaller interlayer distance “*d*”, indicating the energetic feasibility of stacking (d) vdW heterostructure.

**Fig. 3:**
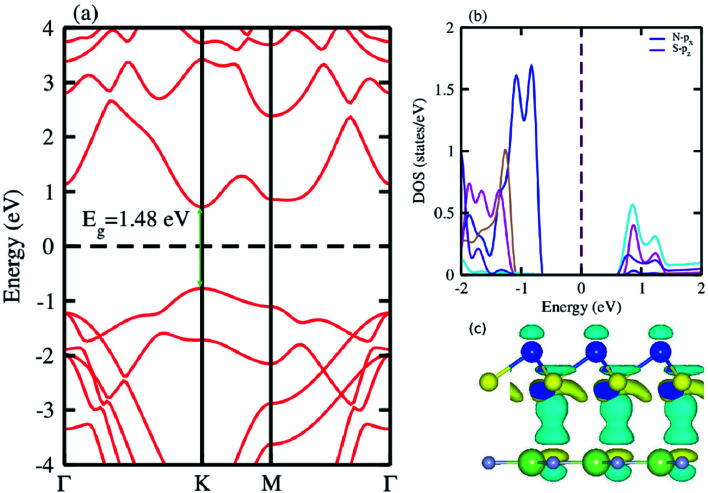
(a) Electronic band structure calculated using PBE functional; (b) partial density of states (PDOS); (c) the 3D isosurface of the charge density difference of the GaN–SiS vdW heterostructure.

**Fig. 4:**
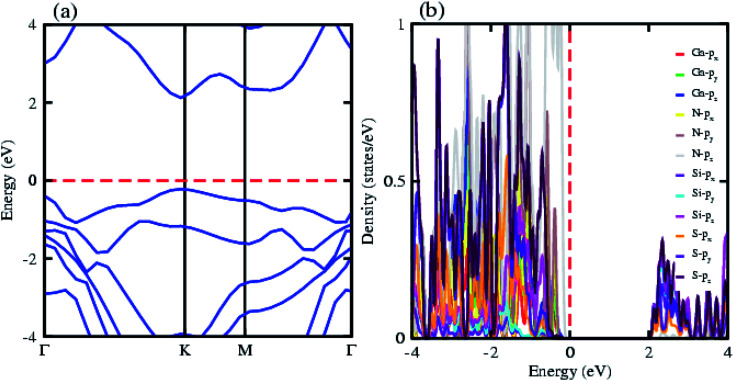
(a) Electronic band structure calculated using HSE06 functional; (b) partial density of states (PDOS) of the GaN–SiS vdW heterostructure.

The electronic band structure of the most feasible configuration of the GaN–SiS vdW heterostructure was calculated using the PBE functional, as shown in Fig. 3(a). The GaN–SiS vdW heterostructure exhibited a direct bandgap (1.48 eV) with VBM and CBM located at the *K*-point of the Brillouin zone. The GaN–SiS vdW heterostructure had a smaller band gap than the pristine GaN and SiS monolayers. However, it exhibited a considerably larger band gap suitable for a photocatalytic reaction, indicating that the GaN–SiS electronic structure could be used as a visible light photocatalyst.^64^ For an accurate description of the band gap, we have calculated the HSE06 band structure for the GaN–SiS vdW heterostructure, which also exhibited a direct band nature with a band gap value of 2.45 eV (see Fig. 4(a)).

**Fig. 5:**
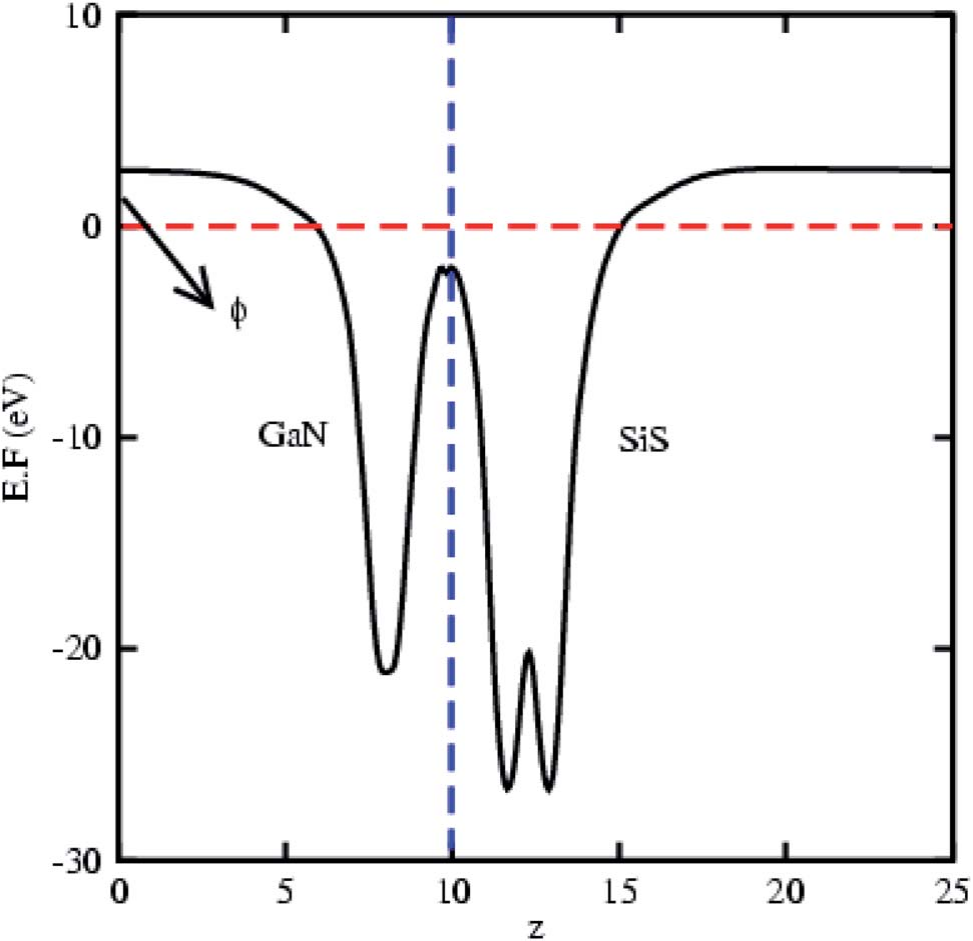
Average electrostatic potential and work function of the GaN–SiS vdW heterostructure.

The partial density of states of the vdW heterostructure of GaN–SiS, as presented in Fig. 3(b), is crucial for understanding the band alignment. It is clear that VBM is contributed by the p_*y*_ orbital of the Si atom, while CBM is mainly dominated by the p_*x*_ orbital of the N atom, as shown in Fig. 3(b). This confirms the type-II band alignment, which efficiently separates electrons and holes in different constitutions. Moreover, some other contributions of Ga and S atoms are also present (see Fig. 4(b)). The interlayer charge transfer is shown by the charge density difference, as displayed in Fig. 3(c). Hence, the charge density difference Δ*ρ* = *ρ*_GaN−SiS_ − *ρ*_GaN_ − *ρ*_SiS_ of the GaN–SiS vdW heterostructure was investigated, where *ρ*_GaN−SiS_ is the charge density of the heterostructure, and *ρ*_GaN_ and *ρ*_SiS_ are the charge densities of isolated GaN and SiS monolayers, respectively. The yellow color represents electron depletion, while the cyan areas show accumulation (Fig. 3(c)). The SiS layer donates electrons to the GaN layer, thus causing the SiS layer to behave as a p-doped layer. In addition, Bader charge analysis was used to estimate the total number of electrons transferred at the interface of GaN–SiS. An amount of 0.0235|*e*| charge was transferred from SiS to GaN layer, thus indicating p-type doping in the SiS monolayer.^65,66^ The charge redistribution was mainly found at the interface, which was responsible for the built-in electric field and separates photogenerated charge carries in different layers of the GaN–SiS heterostructure. This process indicates weak interaction between the GaN and SiS layers. Furthermore, we calculated the average charge density and work function of the GaN–SiS vdW heterostructure. The average charge density showed that the SiS layer has a higher potential than GaN, hence confirming the charge transfer (see Fig. 5). The calculated work function value for the GaN–SiS vdW heterostructure was 3.8 eV.

**Fig. 6:**
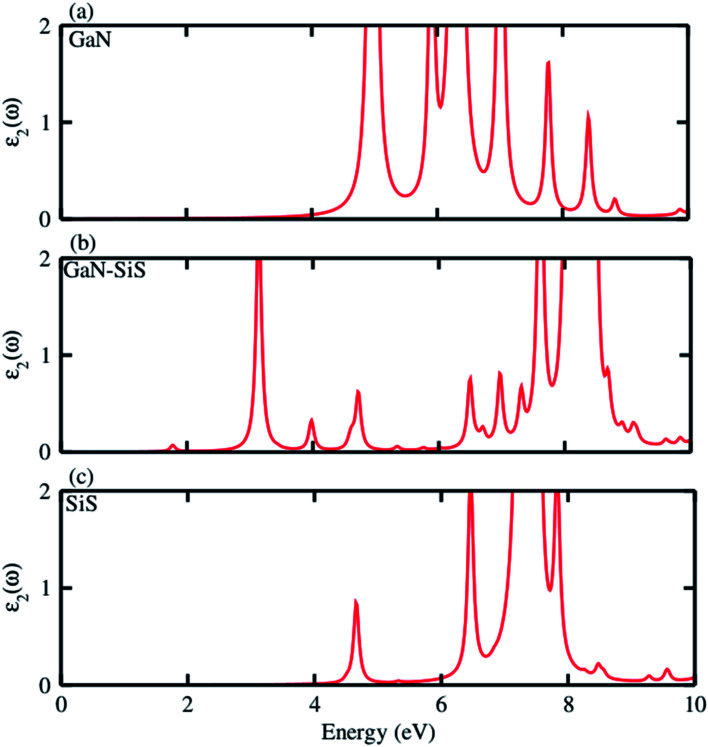
Imaginary part *ε*_2_(*ω*) of the dielectric function of the (a) GaN monolayer, (b) GaN–SiS vdW heterostructure and (c) isolated SiS monolayer.

Further, the absorption spectra in terms of the imaginary part of the dielectric function *ε*_2_(*ω*) was determined for the GaN, SiS monolayers and GaN–SiS heterobilayer, as shown in Fig. 6(a–c). It is clear from Fig. 6(a and c) that exciton peaks are found at 4.97 eV and 4.65 eV in the GaN and SiS monolayers, respectively. In contrast to the parent monolayers, the excitonic peaks shifted to lower energy and were found at 1.77 eV in the GaN–SiS system. This excitonic shift will affect the effective separation of photogenerated electron–hole pairs, which directly influence the efficiency of photocatalytic water splitting. Evidently, a red-shift was found in GaN–SiS as a result of a lower band gap of the GaN–SiS heterobilayer than the corresponding monolayers;^67,68^ hence, GaN–SiS may be a potential candidate for optoelectronic applications. A similar trend was also found in SiC–TMDC and WS_2_–MoS_2_ heterostructures.^69^

**Fig. 7:**
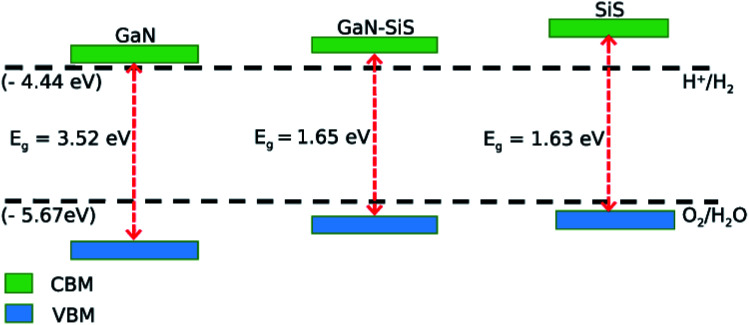
Valence and conduction band edges of GaN and SiS monolayers, and the GaN–SiS vdW heterostructure.

Mulliken electronegativity was used to study the photocatalytic behavior of the GaN–SiS heterostructures for water splitting.^70,71^*E*_CBM_ = *E*_VBM_ − *E*_g_*E*_VBM_ = *χ* − *E*_elec_ + 0.5*E*_g_In the above equations, *χ* is the geometric mean of constituent atoms, while *E*_elec_ is the standard electrode potential having a numerical value of 4.5 eV on the hydrogen scale. Band gap values obtained using the PBE functional was still greater than 1.23 eV, which is the minimum energy required for photocatalysis. This suggested that both GaN and SiS monolayers, and the GaN–SiS heterostructure may be suitable for photocatalytic water splitting under irradiation of solar light, as shown in Fig. 7. The conduction and valence band edges of both the GaN and SiS monolayers, and the GaN–SiS heterobilayer occurred at an energetically stable position and straddled the standard redox potentials, making them promising for water decomposition at pH = 0. This is crucial for photocatalytic hydrogen generation under sunlight. A similar trend has also been demonstrated in GeC*–*MSSe and SiC–TMDCs.^66,68^ Thus, the GaN–SiS vdW heterostructure and corresponding monolayers are predicted as potential candidates for renewable and clean energy applications.^72^

## 4. Conclusion

First principles calculations were performed to explore different properties including, structural, electronic, optical and photocatalytic properties of the GaN–SiS vdW heterostructure. The most feasible configuration was energetically stable. Both the GaN and SiS possessed indirect semiconducting band gap nature. The understudy heterobilayer exhibited direct type-II band alignment, and the photogenerated electrons and holes were spatially separated, which is crucial for solar cell device applications. The charges were transferred from SiS to the GaN monolayer, and SiS became p-type doping in the GaN–SiS vdW heterostructure. Furthermore, a systematically red-shift with lower energy excitonic transition was found in the corresponding vdW heterostructure. Interestingly, the valence band and conduction band edges of both heterobilayer and pristine monolayers are were located above and below the standard redox potentials, for photocatalytic water decompositions, which suggested that the understudy monolayers and heterobilayer systems were meant for water dissociation under a solar spectrum. The findings open up an avenue for designing new devices based on the GaN–SiS heterobilayer for promising optoelectronic and photocatalytic water splitting to produce hydrogen as a renewable energy source.

## Conflicts of interest

There are no conflicts of interest to declare.

## Supplementary Material
