# Correction: First principles study of electronic and optical properties and photocatalytic performance of GaN–SiS van der Waals heterostructure

**DOI:** 10.1039/d3ra90031b

**Published:** 2023-04-13

**Authors:** S. S. Sabir, M. Farooq, H. U. Din, Q. Alam, M. Idrees, M. Bilal, B. Amin

**Affiliations:** a Department of Physics, Hazara University Mansehra Pakistan; b Department of Physics, Abbottabad University of Science and Technology Abbottabad 22010 Pakistan haleem.uddin@yahoo.com; c Department of Physics, Bacha Khan University Charsadda Pakistan

## Abstract

Correction for ‘First principles study of electronic and optical properties and photocatalytic performance of GaN–SiS van der Waals heterostructure’ by S. S. Sabir *et al.*, *RSC Adv.*, 2021, **11**, 32996–33003, https://doi.org/10.1039/D1RA06011B.

The authors regret that the name of the first author (Shah Saleemullah Sabir) was shown incorrectly in the original article. The corrected author list is as shown above.

The Royal Society of Chemistry apologises for these errors and any consequent inconvenience to authors and readers.

## Supplementary Material

